# Phytochemical Analysis and Antioxidant and Antifungal Activities of Powders, Methanol Extracts, and Essential Oils from *Rosmarinus officinalis* L. and *Thymus ciliatus* Desf. Benth.

**DOI:** 10.3390/ijms25147989

**Published:** 2024-07-22

**Authors:** Noui Hendel, Djamel Sarri, Madani Sarri, Edoardo Napoli, Antonio Palumbo Piccionello, Giuseppe Ruberto

**Affiliations:** 1 Department of Microbiology and Biochemistry, Faculty of Sciences, University Mohamed Boudiaf of M’sila, M’sila 28000, Algeria; 2 Laboratory of Biology: Applications in Health and Environment, University of M’sila, M’sila 28000, Algeria; 3 Department of Nature and Life Sciences, Faculty of Sciences, University Mohamed Boudiaf of M’sila, M’sila 28000, Algeria; djamel.sarri@univ-msila.dz (D.S.); madani.sarri@univ-msila.dz (M.S.); 4 Institute of Biomolecular Chemistry, National Research Council ICB-CNR, 95126 Catania, Italy; edoardo.napoli@icb.cnr.it (E.N.); giuseppe.ruberto@icb.cnr.it (G.R.); 5 Dipartimento di Scienze e Tecnologie Biologiche, Chimiche e Farmaceutiche-STEBICEF, Università degli Studi di Palermo, Viale delle Scienze Ed. 17, 90128 Palermo, Italy; antonio.palumbopiccionello@unipa.it

**Keywords:** essential oils, chemical composition, polyphenolic contents, *Rosmarinus officinalis*, *Thymus ciliatus*, antioxidant activity, antifungal activity

## Abstract

Chemical residues in food pose health risks such as cancer and liver issues. This has driven the search for safer natural alternatives to synthetic fungicides and preservatives. The aim of this study was to characterize the chemical composition of the essential oils (EO), determine the polyphenolic contents, and evaluate the in vitro antioxidant and antifungal activities of methanol extracts (ME), essential oils (EO), and powders from *Rosmarinus officinalis* L. (rosemary) and *Thymus ciliatus* (Desf) Benth. (thyme) from the M’sila region, Algeria. The chemical composition of the EOs was determined by GC-MS. *R. officinalis* EO was composed of 31 components, mainly camphor (41.22%), camphene (18.14%), and α-pinene (17.49%); *T. ciliatus* EO was composed of 58 components, mainly, in percentage, α-pinene (22.18), myrcene (13.13), β-pinene (7.73), β-caryophyllene (10.21), and germacrene D (9.90). The total phenols and flavonoids were determined spectrophotometrically, and the rosemary ME was found to possess the highest polyphenolic content (127.1 ± 2.40 µg GAE/mg), while the thyme ME had the highest flavonoid content (48.01 ± 0.99 µg QE/mg). The antioxidant activity was assessed using three methods: rosemary ME was the most potent, followed by DPPH (IC_50_ = 13.43 ± 0.14 µg/mL), β-carotene/linoleic acid (IC_50_ = 39.01 ± 2.16 μg/mL), and reducing power (EC_50_ = 15.03 ± 1.43 µg/mL). Antifungal activity was assessed for 32 pathogenic and foodborne fungi. Four methods were applied to the solid medium. Incorporating the powdered plant into the culture medium (at 10%) reduced the fungal growth to greater than 50% in 21.88% and 6.25% of all fungal isolates, for *R. officinalis* and *T. ciliatus*, respectively. The ME, applied by the well diffusion method (0.1 g/mL), was less effective. Different concentrations of EO were tested. Incorporating the EO into the culture medium (1500 μL/L) inhibited 50% of the molds to levels of 50 and 75% for *R. officinalis* and *T. ciliatus*, respectively, with the complete inhibition of four fungi. Fumigated EO (15 μL) inhibited 65% of the molds to levels of 65 and 81.25% for *R. officinalis* and *T. ciliatus*, respectively, with the complete inhibition of five fungi. There was little to no sporulation in conjunction with the inhibition. Our results revealed some of the potential of the studied plants to fight foodborne molds and presented their promising characteristics as a source of alternatives to chemical pesticides and synthetic preservatives. Further studies are needed to find adequate application techniques in the food safety area.

## 1. Introduction

Plants produce antioxidant molecules such as vitamins and phenolic compounds, which have beneficial effects on human health [1,2]. Natural-origin compounds have multiple advantages. Numerous investigations have exhibited their expanding uses as immune-modulatory, sedative, analgesic, antioxidant, antibacterial, and anticancer drugs [3,4]. They might occasionally be safer options for antibiotics [5]. 

Molds are able to grow on all kinds of foods: cereals, meat, milk, fruit, vegetables, nuts, and fats. Their growth can lead to several types of food disorders: abnormal flavors, toxins, discoloration, rot, and the formation of pathogenic or allergenic propagules [1]. The most important aspect that accompanies economic losses due to food deterioration by molds is the formation of mycotoxins. More than 500 mycotoxins are already known, with aflatoxins being the most well-known. There are three main families of molds that have an impact on human health: *Aspergillus*, *Penicillium*, and *Fusarium* [6,7]. Synthetic chemicals used as food preservatives have been linked to negative health consequences in humans, including allergies and cancer. Using synthetic fungicides may also lead to the development of resistant fungal strains [8]. 

Some essential oil components are used as food flavoring additives and are associated with some biological properties, such as antioxidant and antimicrobial activity, among others. These actions can resolve numerous problems, including resistance to antimicrobials [9]. 

Rosemary (*Rosmarinus officinalis* L.), also called k’lil or azir in Algeria, is an evergreen perennial small shrub belonging to the Lamiaceae family and is very branched and abundantly thick with a characteristic aromatic smell. It grows most often in scrubland and pine, cedar, or juniper forests. It is found in various bioclimates (from sub-humid to higher arid), but it is dominant in the semi-arid Mediterranean bioclimate areas [10]. Originating in the Mediterranean region, rosemary is now grown around the world. The parts often used are fresh or dried leaves that are whole, cut, crushed, or grated, and essential oil [11]. This aromatic plant is used in cooking as a flavor for meat, fish dishes, rice, and salads, as well as in folk medicine for the treatment of digestive and liver disorders and scalp and skin conditions. It is also known as an antioxidant, antimicrobial, antibiofilm, anti-inflammatory, digestive, and tonic [12,13,14,15,16]. Rosemary was recently the subject of studies on its antifungal, antiaflatoxigenic, and herbicidal effects [17,18,19,20].

*Thymus ciliatus* (Desf.) Benth., belonging to the Lamiaceae family, locally called *Djertil*, is a raised or prostrated odorous shrub. This plant, which is endemic to North Africa, has important biological activities: antifungal, antibacterial, antiviral, and antioxidant. Thyme is used in traditional medicine for its antiseptic, antispasmodic, diaphoretic, stimulant, antitussive, sedative, and ruby effects [21,22,23,24]. To the best of our knowledge, few studies have been performed on the antifungal activity of *Thymus ciliatus* [24,25].

This work is part of the valorization of the local flora of therapeutic interest, with the aim of evaluating *Rosmarinus officinalis* and *Thymus ciliatus* in terms of EO composition, testing their powders, methanol extracts, and EO for total phenolics and antioxidant activity, and testing their antifungal effects on some members of the phytopathogenic fungal flora and other mycotoxin producers.

## 2. Results and Discussion

### 2.1. Extraction Yields

According to the applied operating mode and based on dry matter weight calculation (*w*/*w*), the methanol extraction resulted in 30.87 ± 3.2% and 27.14 ± 1.8% for *R. officinalis* and *T. ciliatus*, respectively.

In general, several factors influence EO yields, including plant variety, ecological characteristics of the harvest area, harvest time, and extraction method, among others [26,27]. The EO yields for our plants were 1.14 ± 0.15 and 1.5 ± 0.1% (*v*/*w*) for *R. officinalis* and *T. ciliatus*, respectively. In comparison to other authors, El Kamli et al. [28] mentioned yield values of 2.14–2.25% (*v*/*w*) from Moroccan rosemary; Cutillas et al. [29] mentioned 0.8–1.1% as the yield of rosemary from Spain, and rosemary from Tunisia yielded 1.3–1.69 (*w*/*v*) [30]. *T. ciliatus* from North Africa has EO yields that also vary according to the region and the harvest period, from 0.3% to extreme levels of 5.1% [31,32,33], with median levels of 2–3% [24,34].

### 2.2. GC and GC-MS Analyses of the EOs

Table 1 shows the composition of the EOs of the studied plants. The EO constituents have been characterized and grouped into three categories: monoterpene hydrocarbons, oxygenated monoterpenes, and sesquiterpenes. For *R. officinalis*, 31 compounds (97.03% of the total) were annotated. The content of monoterpene hydrocarbons and oxygenated hydrocarbons was very high compared to that of sesquiterpenes; their values were 43.97, 50.09, and 2.97%, respectively. The EO is characterized by the predominance of certain components, namely camphene (18.14%) and α-pinene (17.49%) among monoterpene hydrocarbons; camphor (41.22%) and 1,8-cineole (4.90%) among oxygenated monoterpenes; and α-bisabolol (1.15%) among sesquiterpenes. In a study on 15 samples of rosemary from different regions of Algeria, Hendel et al. [35] showed that the rosemary EO is basically composed of α-pinene (15–21%), camphene (15–22%), limonene (3–5%), camphor (34–41%), 1,8-cineole (2–9%), and borneol (1-4%). Recently, in a study on the Saharan Algerian rosemary EO, it was mentioned that the main components include 1,8-cineole, camphor, borneol, α-pinene, β-pinene, linalool, and verbenone, respectively [15]. According to Cutillas et al. [29], Spanish rosemary is mainly composed of α-pinene (14–28%), camphene (4.8–13%), β-pinene (3.3–6.5%), β-myrcene (1.2–3.6%), limonene (1.9–5.2%), cineole (24.7–49.9%), and camphor (10–19.8%). Rosemary from Tunisia is mainly composed of α-pinene (7.09–13.66%), camphene (3.09–5.07%), β-pinene (3.26–3.81%), cineole (46.8–57.88%), camphor (9.27–18.99%), and borneol (4.49–13.21%) [17,30]. Moroccan Rosemary has the main components of α-pinene (15.82%), camphene (9.77%), β-pinene (8.58%), cineole (51.77%), camphor (22.31%), and α-Terpineol (7.36) [36,37].

For *T. ciliatus*, 58 compounds (99.65% of the total) were annotated. The content of monoterpene hydrocarbons and sesquiterpenes was very high compared to the oxygenated monotherpenes: 53.11, 39.33, and 7.16%, respectively. The presence of a non-terpenoid compound, 3-octanone, was noted in very small quantities. Of the 16 monoterpene hydrocarbon components (53.11%), three were mainly predominant, α-pinene (22.18%), myrcene (13.13%), and β-pinene (7.73%), respectively. Oxygenated monoterpenes were minor constituents with 16 compounds (7.16%). Sesquiterpenes (32 components; 39.33%) contained significant amounts of β-caryophyllene (10.21%) and germacrene D (9.90%). It should be noted that thymol and carvacrol were completely absent, even at trace levels; these are monoterpene phenols that are present in many species of *Thymus*. In *Thymus ciliatus* from the neighboring region (Djelfa), only 25 components were identified at up to 97.7% of the EO, with myrcene, p-cymene, and borneol as the main components [38]. Moroccan thymus EOs were analyzed by Jamali et al. [39], and monoterpene hydrocarbons and oxygenated monoterpenes formed the main classes. Chemotaxonomical analysis has allowed classification into three main groups: *T. ciliatus* was found to be in the group containing thymol and/or carvacrol, γ-terpinene, and p-cymene. In the literature, *Thymus* is considered a genus carrying thymol and/or carvacrol [40,41,42,43,44], and Kabouche et al. [45] consider *T. ciliatus* EO to be the EO of the entire genus *Thymus*, containing the highest amount of thymol. However, Ghorab et al. [32] cited an EO from a *T. ciliatus* sample collected in Algeria without thymol or carvacrol and mentioned that the presence and content of thymol vary depending on geographic zone, climate, and soil nature. Recently, Souadia [38] indicated variation in *T. ciliatus* EO thymol content from 0.3% (April) to trace (May) and at trace levels during the flowering stage. He claimed that the EO’s chemical composition, both in terms of quality and quantity, is greatly influenced by the picking location, time, and weather.

### 2.3. Total Polyphenols and Flavonoids

Phenolic compounds are considered secondary ubiquitous metabolites in plants. Flavonoids are naturally found in plants and are considered to have positive effects on human health [46]. Table 2 shows the total polyphenol and flavonoid contents of the MEs and EOs of the studied plants. The content of polyphenolic compounds (µg gallic acid equivalents/mg extract: μg GAE/mg) ranges from a minimum of 7.81 ± 0.41 for the EO of rosemary to a maximum of 127.1 ± 2.40 for the ME of the same plant, whereas the flavonoid content (µg of quercetin equivalents/mg extract: μg QE/mg) is highest for the thyme ME (48.01 ± 0.99). The EOs are poor in polyphenols and flavonoids compared to the extracts. Yesil-Celiktas et al. [47] found polyphenolic levels ranging from 147.3 to 34.1 mg GAE per g of rosemary extract harvested from different areas of Turkey and at different harvest periods. Other studies have shown lower levels of phenolic compounds [48,49,50]. Our Thyme had a higher polyphenolic level than that from western Algeria harvested in May, which contained 64.23 mg GAE/g [22]; this may be due to the ecological characteristics of the areas and periods of harvest or the nature of the plant itself. A study of the phenolic content of EOs in different rosemary clones from different geographical areas in Europe and North Africa showed that the lowest-yield plant EO produced the highest level of polyphenols [51], which explains that the quality of EO is not dependent on the yield.

### 2.4. Antioxidant Activity

#### 2.4.1. DPPH Radical Scavenging Activity Assay

Table 3 shows the IC_50_ values of the MEs and EOs of the studied plants and those of the synthetic antioxidant butylated hydroxytoluene (BHT). The MEs showed a significantly higher scavenger effect than BHT (*p* < 0.05). The EOs were less effective. These results show that our plants have a very interesting antioxidant potential. Thyme showed higher anti-radical activity than the same plant from western Algeria [22] and even higher than other species such as *Thymus pallescens*, *T. algeriensis*, and *T. dreatensis* (IC_50_ = 235–900 μg/mL) [52,53,54]. The EO appears to have lower effectiveness than other sub-species of the same genus: *T. sipyleus* (IC_50_ = 220 ± 0.5 μg/mL (0.27 μL/mL) and 2670 ± 0.5 μg/mL), cited by Tepe et al. [55], and *T. vulgaris* L. (IC_50_ = 189 ± 2.38 μg/mL), cited by Miladi et al. [56]. Compared to other studies [57,58]., our rosemary extract showed high DPPH free radical scavenging activity. The EO showed lower activity compared to that mentioned by Miladi et al. [56] (IC_50_ = 437 ± 5.46 μg/mL). Ojeda-Sana et al. [59] found a high trapping capacity associated with a myrcene chemotype rosemary EO, but Wang et al. [60] and Hussain et al. [61] found greater *R. officinalis* EO activity than its main components 1,8-cineole, camphor, and α-pinene. The latter form the main components of our rosemary EO. 

#### 2.4.2. β-Carotene Bleaching Test

The results obtained from the beta-carotene bleaching test showed an order of effectiveness similar to that shown in the DPPH free radical scavenging test (Table 3). Nevertheless, the MEs IC_50_ values were higher when compared with BHT, which showed a strong inhibition of low-concentration β-carotene bleaching. The EO of *T. ciliatus* appears to be more effective (2.21 mg gave an inhibition of 91.38%) compared to that of the same plant (87.39% was measured at 4 mg/mL), as cited by Ghorab et al. [62]. Other species of the *Thymus* genus showed higher activity, ranging from 23.62 to 92.87 μg/mL [63].

#### 2.4.3. Reducing Power Test

As shown in Table 3, the EC_50_ values showed reducing power in the order of BHT > MER > MET > EOT > EOR. In this test, the EO of the studied thyme is weak compared to those of other spontaneous or cultivated species [64].

The plant phenolic hydroxyl groups have strong trapping capacity, and the flavonoids possess potential antioxidant activities [46]. Apolar antioxidants may exhibit stronger antioxidant properties in emulsions as they concentrate in the lipid phase, and polar antioxidants remain in the aqueous phase and are therefore less effective in the protection of lipids [64]. The composition of rosemary extract varies depending on the type of sample, the location, and the time of harvest, so different extracts from different geographical areas and different time points also vary considerably [48]. The antioxidant activity of rosemary may be related to rosmarinic acid, carnosol, rosmanol, carnosic acid, and the phenolic compounds of rosemaridiphenol [65]. Rosemary may have a synergistic effect with other natural antioxidants; this synergy would allow better food preservation by delaying lipid oxidation [66]. The genus *Thymus* has two main groups of secondary metabolites: volatile terpenes and polyphenolic compounds. Both are primarily responsible for such biological effects as antioxidant activities [11].

### 2.5. Antifungal Activity

The antifungal activity was evaluated by testing the plant as a powder in the culture medium, testing the EOs by fumigation and incorporation into the culture medium, and testing the MEs using the well-diffusion method. The radial growth of each tested fungus was measured daily and compared to that of the control. The inhibition percentage was calculated on the 7th day for all applied techniques.

In general, all the concentrations applied by the different methods have significantly reduced or completely inhibited the growth of the tested molds. 

**Figure 1:**
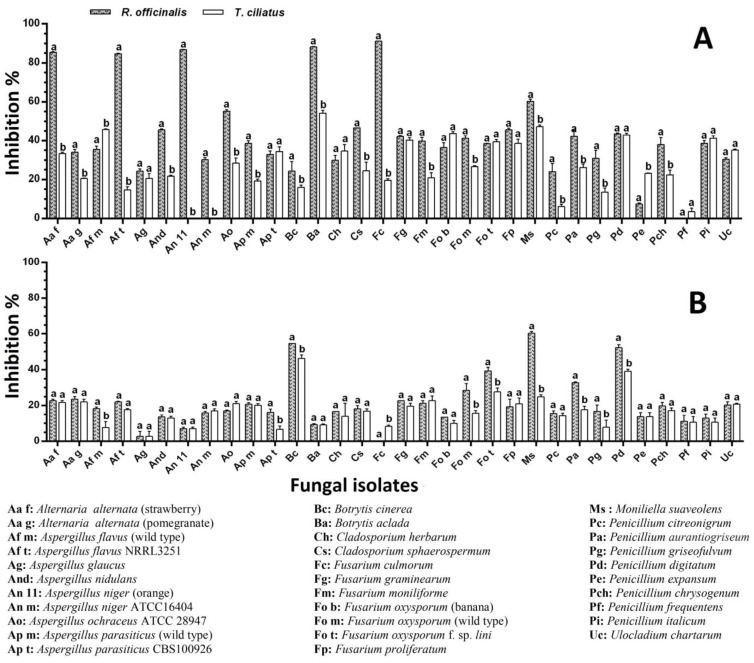
Effect of powders and methanol extracts of *R. officinalis* and *T. ciliatus* on the radial growth of tested molds grown on Potato Dextrose Agar (PDA). (**A**) Powder (10%, *w*/*v*); (**B**) methanol extract (0.1 g/mL). The data are represented as the average ± SD (*n* = 3). Different letters indicate significant differences (*p* < 0.05) between the two tested plants on each mold, according to Sidak’s multiple comparisons test.

**Figure 2:**
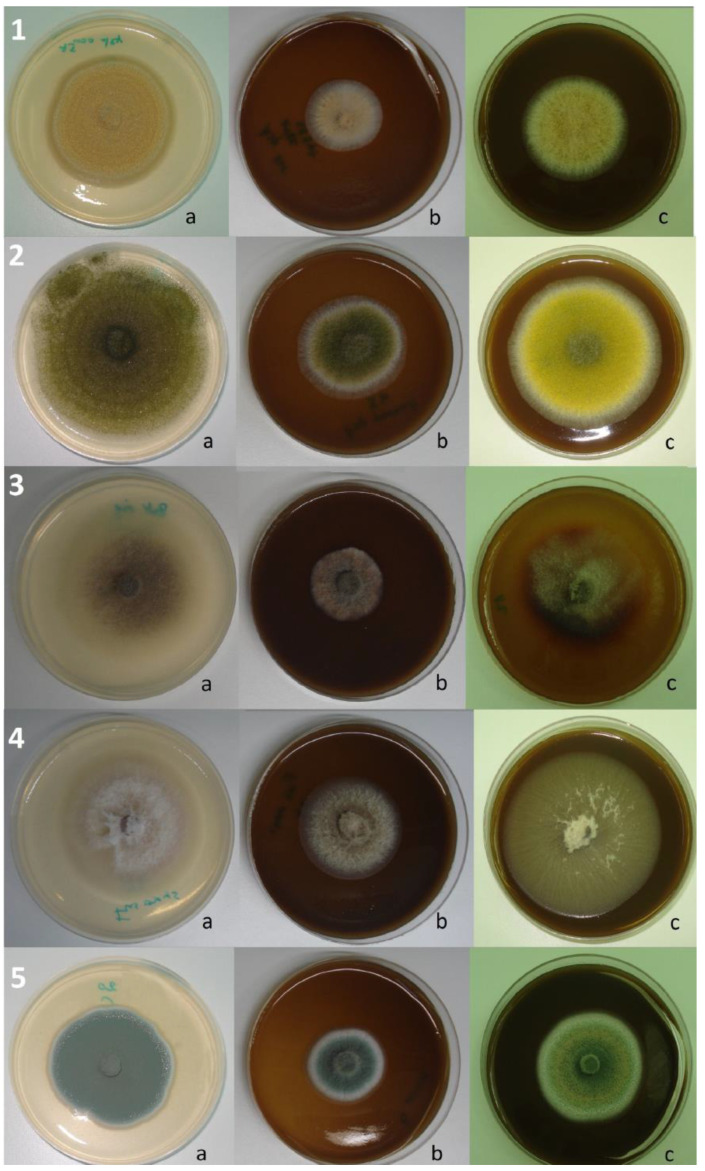
Effect of the powdered plant on the radial growth of some tested fungi; 1—*A. ochraceus*, 2—*A. parasiticus*, 3—*B. aclada*, 4—*F. oxysporum*, 5—*P. expansum*; (a) control, (b) PDA medium supplemented with *R. officinalis*, and (c) PDA medium supplemented with *T. ciliatus*.

*R. officinalis* applied as an embedded powder (10%, *w*/*v*) showed more than a 10% inhibitory effect on 30 isolates (93.75% of the total number); 7 of these (21.88% of the total) were inhibited to a level of 50–90%. At the same time, *T. ciliatus* showed the inhibition of more than 10% of 28 isolates (87.5% of the total number), of which 2 (6.25%) were inhibited to a level of 50–60% (Figure 1A). Compared to controls, the molds subjected to the powder effect showed a more or less different cultural aspect; some fungi have undergone a change in colony color (*A. parasiticus*, *P. chrysogenum*, *A. nidulans*, etc.), the restriction of margins (*A. alternata*), or sometimes an abundance of aerial mycelium with sporulation restriction (*A. flavus*), or even the appearance of coremia and the absence of superficial exudates (*P. expansum* and *P. aurantiogriseum*, etc.) (Figure 2). The statistical analysis showed no significant difference (*p <* 0.05) between the effects of the two plants on 11 strains (34.37% of the total), according to Sidak’s Multiple Comparisons Test.

**Figure 3:**
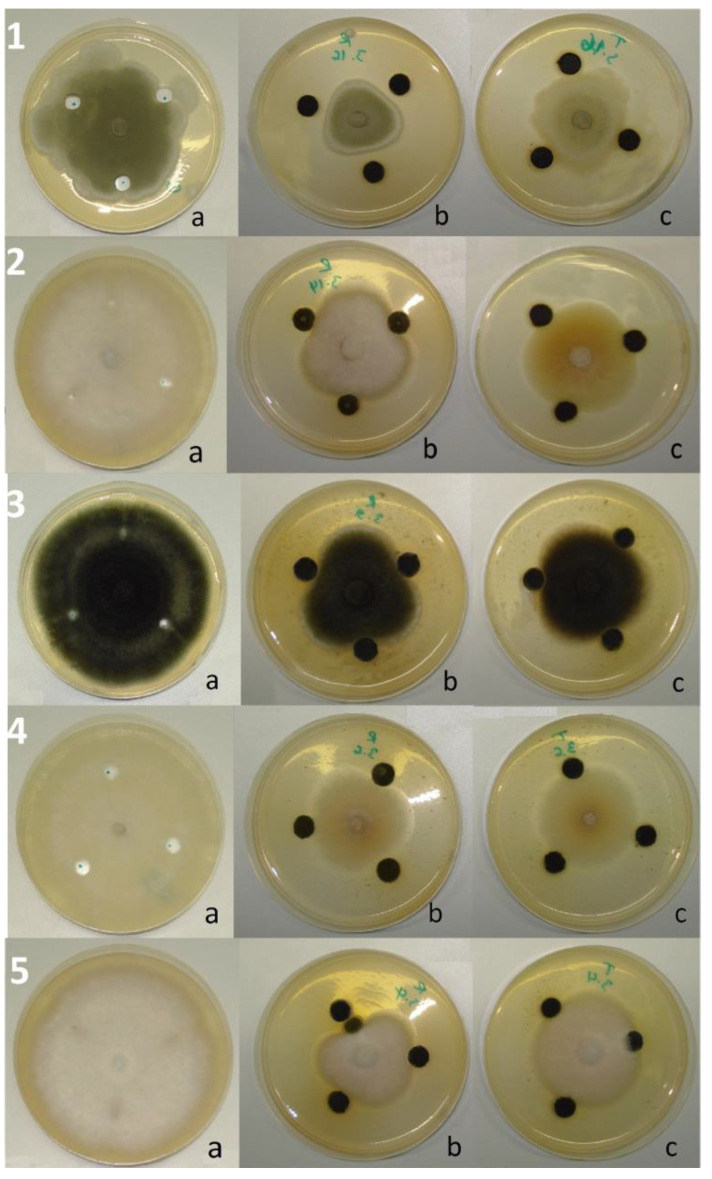
Effect of the plant ME on the radial growth of some tested fungi; 1—*P. digitatum*, 2—*F. graminearum*, 3—*A. alternata*, 4—*F. oxysporum*, 5—*F. proliferatum*; (a) control, (b) PDA medium supplemented with *R. officinalis*, (c) PDA medium supplemented with *T. ciliatus*.

The ME of *R. officinalis* applied by the well technique (at 0.1 g/mL) showed an inhibition level of more than 10% out of 28 fungal isolates (87.50% of the total number). Three of these (9.38% of the total) were inhibited at 50–70%. At the same time, *T. ciliatus* ME showed an inhibition level of more than 10% on 24 isolates (75% of the total), with a maximum degree of around 50% (Figure 1B). Compared to the controls, the isolates subjected to the effect of ME showed sporal insufficiency to varying degrees from one mold to another, with the appearance of pigmented secretions in the culture medium and restriction of the margin of the colonies (Figure 3). The statistical analysis showed no significant difference (*p* < 0.05) between the effects of the two plant MEs on 22 isolates (68.75% of the total), according to Sidak’s Multiple Comparisons Test.

**Figure 4:**
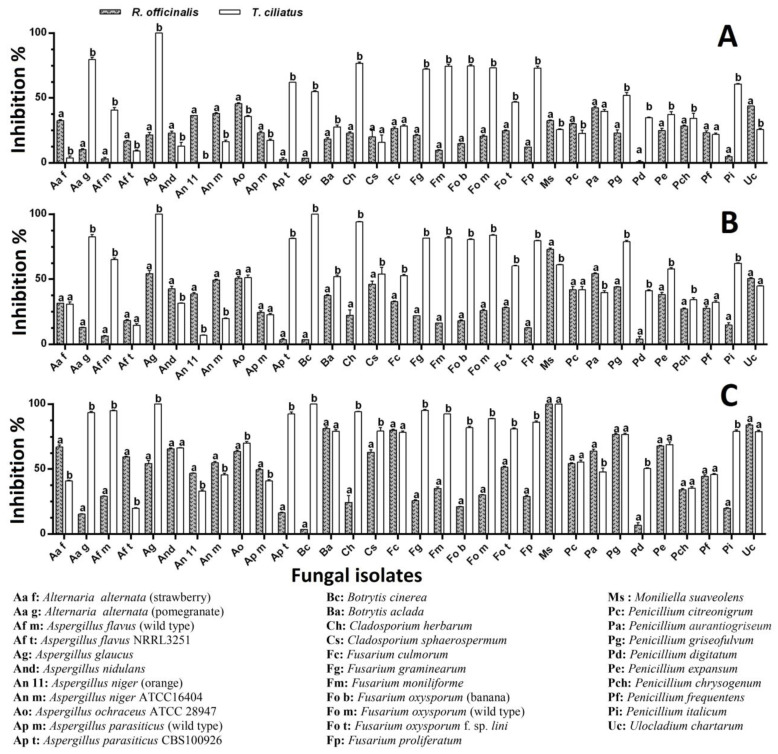
Effect of EOs of *R. officinalis* and *T. ciliatus* on the radial growth of the tested molds grown on PDA by direct contact method of (**A**) 500, (**B**) 1000, and (**C**) 1500 μL/L. The data are represented as the average ± SD (*n* = 3). Different letters indicate significant differences (*p* < 0.05) between the two tested plants on each mold, according to Sidak’s multiple comparisons test.

The *R. officinalis* EO applied by the incorporation of increasing concentrations in the culture medium caused inhibition of more than 10% of 81.25, 87.50, and 93.75% of the fungal isolates at concentrations of 500, 1000, and 1500 μL/L, respectively. Fifty percent (50%) of the isolates underwent inhibition ranging from 50% to 100% at the higher concentration. At the same time, *T. ciliatus* EO showed inhibition of more than 10% of 90.63, 96.88, and 100% of the fungal isolates for the same concentrations (500, 1000, and 1500 μL/L), respectively. Seventy-five percent (75%) of the isolates underwent inhibition ranging from 50% to 100% at the higher concentration. As shown in Figure 4, thyme EO appears to be more effective than rosemary EO; the significant difference (*p <* 0.05) concerned 28 isolates (87.5% of the total), of which 18 (56.25% of the total) were more sensitive to thyme EO (500 μL/L) (Figure 4A); for a concentration of 1000 μL/L, the significant difference concerned 25 isolates (78.13% of the total), of which 20 (62.5% of the total) were more sensitive to the thyme EO (Figure 4B); for the 1500 μL/L concentration, the significant difference concerned 22 isolates (68.75% of the total), of which 16 (50% of all) were most sensitive to thyme EO (Figure 4C).

**Figure 5:**
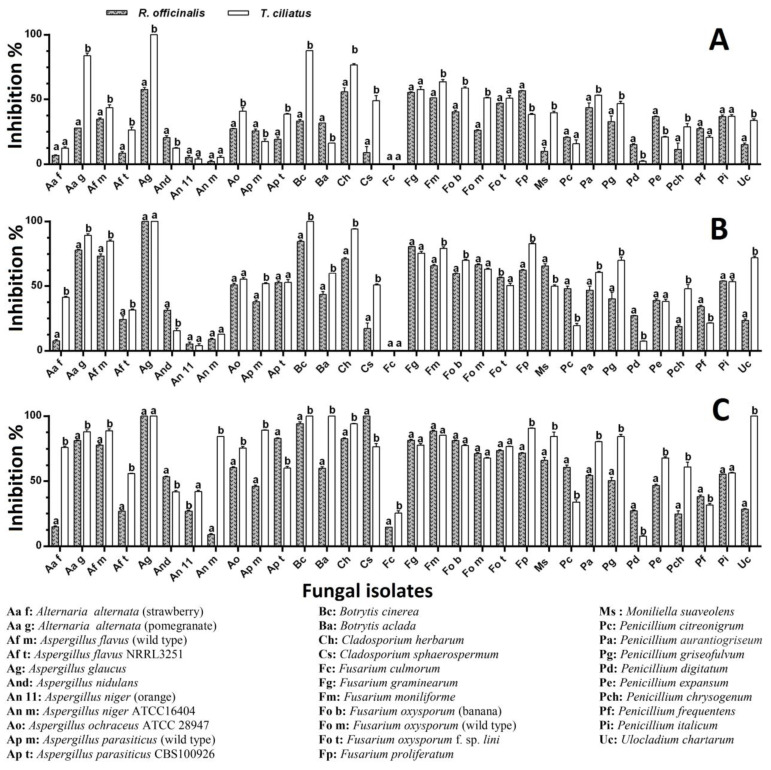
Effect of EOs of *R. officinalis* and *T. ciliatus* on the radial growth of the tested molds grown on PDA by direct contact method of (**A**) 5, (**B**) 10, and (**C**) 15 μL. The data are represented as the average ± SD (*n* = 3). Different letters indicate significant differences (*p <* 0.05) between the two tested plants on each mold, according to Sidak’s multiple comparisons test.

The *R. officinalis* EO applied by fumigation at increasing concentrations (5, 10, and 15 μL/plate) caused inhibition of more than 10% of 78.13, 87.50, and 96.88% of the fungal isolates at volumes of 5, 10, and 15 µL, respectively. A number of 21 isolates (65.63%) underwent inhibition ranging from 50% to 100% at the higher concentration. At the same time, *T. ciliatus* EO showed inhibition of more than 10% of 87.5, 90.63, and 96.88% of the fungal isolates at the same volumes. Twenty (62.5%) and 26 (81.25%) isolates underwent inhibition ranging from 50% to 100% at median and higher concentrations, respectively. As shown in Figure 5, thyme EO appears to be more effective than rosemary EO; the significant difference (*p <* 0.05) concerned 23 isolates (71.88% of the total), among which 17 (53.13% of the total) are more sensitive to the thyme EO (5 μL/plate) (Figure 5A); at the concentration of 10 μL/plate, the significant difference concerned 22 isolates (68.75% of all), of which 16 (50% of the total) were more sensitive to the thyme EO (Figure 5B); at the 15 μL/plate concentration, the significant difference concerned 25 isolates (78.13% of all), of which 19 isolates (59.38% of the total) were most sensitive to the thyme EO (Figure 5C).

**Figure 6:**
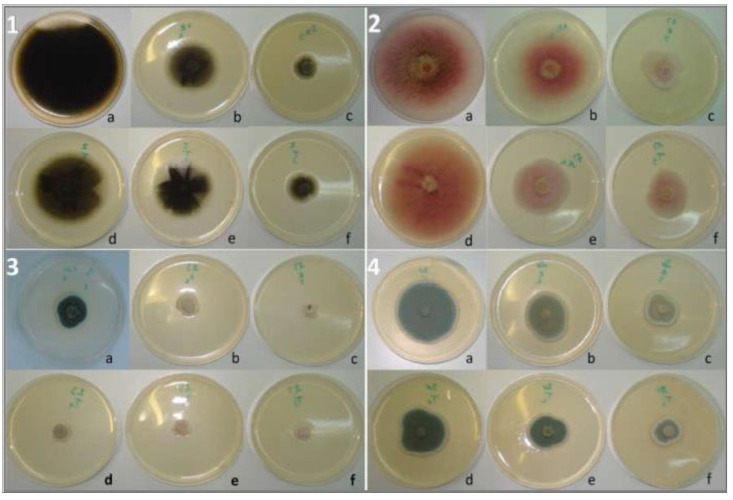
Effect of direct contact with EO on the radial growth of (1) *M. suaveolens*, (2) *F. culmorum*, (3) *P. griseofulvum,* and (4) *P. expansum*; a: Control; b,c: mold exposed to concentrations of 1000 and 1500 µL/mL of *R. officinalis* EO, respectively; d–f: mold exposed to concentrations of 500, 1000, and 1500 µL/mL of *T. ciliatus* EO, respectively.

**Figure 7:**
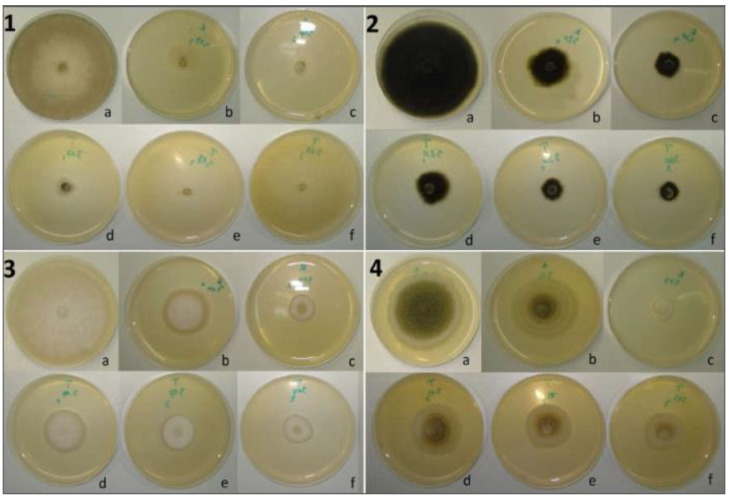
Effect of EO fumigation on the radial growth of (1) *B. cinerea*, (2) *A. alternata*, (3) *F. graminearum,* and (4) *A. flavus*; a: Control; b,c: mold exposed to fumigation of 10 and 15 µL of *R. officinalis* EO, respectively; d–f: mold exposed to fumigation of 5, 10, and 15 µL of *T. ciliatus* EO, respectively.

Compared to the controls, apart from the completely inhibited isolates, the molds subjected to the effect of the two EOs showed a different outcome depending on the cultivation method; the decrease in spore density was effective in the vast majority of the molds, and this was especially evident under the action of EOs at higher concentrations, depending on the applied technique (fumigation or incorporation). The cultivation aspect more or less changes in some isolates, such as *Fusarium* and *Aspergillus* species; the mycelium becomes dense and narrow with contracted margins or is accompanied by the appearance of coremia (*A. parasiticus* and *F. oxysporum*) or dispersed sporulation with condensation of the mycelium at the colony center (*A. niger*, *B. aclada*). Some molds secreted pigments, and others presented superficial exudates (*P. frequentens*, *P. expansum*) (Figure 6 and Figure 7).

Many plants, particularly those belonging to the Lamiaceae family, are known for their antimicrobial activity, especially their EOs. The different methods used revealed that *R. officinalis* and *T. ciliatus* are plants endowed with remarkable antifungal activity. The EOs have proven their effectiveness compared to MEs and powder. The EO vapor technique was the most effective. It was reported that *P. digitatum* growth was inhibited up to 71.4% by vaporized rosemary EO, and the rot on orange fruits treated with 900 ppm was reduced by 12.5%, apart from the inhibition of sporulation [67]. Similarly, the *R. officinalis* EO showed an impressive inhibitory effect on *Fusarium oxysporum* f. sp. *albedinis*. The inhibition on the seventh day was around 24 and 65% via the micro-atmosphere and direct contact methods, respectively [19]. Other studies confirmed the sensitivity of *P. digitatum* to EOs and plant extracts by the type of solvent [25]. Rosemary extracts are known to include the more active compounds carnosic acid, carnosol, and rosmarinic acid. Carnosic acid has been shown to exhibit higher antioxidant and antimicrobial activities. Moreover, these activities were improved in aqueous systems by complexation with flexible cyclic glucans, such as cycloamylose [68]. The examination of the antifungal activity of EOs of *Thymus daenensis* Celak., *Zataria multiflora* Boiss, and *Thymbra spicata* L. against *Aspergillus flavus*, *A. fumigatus*, *A. niger*, and *A. parasiticus* showed strong activity in *Thymus daenensis* Celak compared to the other plants [69]. A study of the antifungal activity of several EOs, applied by microdilution and fumigation techniques, on 44 fungal strains belonging to various genera showed that among the EOs studied, those of *Origanum vulgare* L., *Thymus serpyllum* L., *T. vulgaris*, *Lavandula latifolia* Medik., and *L. angustifolia* inhibited fungal growth. The antifungal action was attributed to phenolic compounds, including carvacrol and thymol [70]. These were the most powerful inhibitors of the fungus *Botrytis cinerea* in vitro [71]. Monoterpenes like camphor and 1,8-cineole also possess antibacterial and antifungal qualities [72]. This antifungal activity may be due to the chemical composition of the EOs and the leaves. The EOs contain important compounds such as α-pinene, bornyl acetate, camphor, rosmarinic acid, 1.8 cineole, thymol, carvacrol, γ-terpinene, and p-cymene [73]. Wang et al. [74] mentioned that natural borneol inhibited *C. albicans* in both the vapor and liquid phases and also reduced the yeast biofilm activity by up to 58.2%, and the effect was dose-dependent. Da Silva Bomfim et al. [75] found that the application of the *R. officinalis* EO at 150–600 µg/mL concentrations to *Fusarium verticillioides* reduced microconidia production and caused the apparent rupture of the cell wall and leakage of the cytoplasmic contents through the loss of membrane integrity and blockage of cell growth. Likewise, *R. officinalis* EO affected the spore production and reduced the thickness of the hyphae of *A. flavus*; furthermore, there was a significant decrease in the ergosterol content [18].

The mode of action of EOs on microorganisms is not clearly determined, and their antimicrobial activity has been shown to be dependent on their hydrophobicity and partition in microbial membranes. This eventually causes cell death by allowing vital chemicals to seep out [76]. In general, EOs cause damage to microbial structures and functions by disrupting membrane permeability and the osmotic balance of the cell. Phenolic compounds play a major role [20,77,78]. Studies on the antimicrobial properties of thyme EO particularly attribute this action to phenolic compounds like thymol and carvacrol. Lambert et al.’s [79] study of the EOs of oregano and two of its main constituents, thymol and carvacrol, against bacteria suggests that these compounds alter the integrity of bacterial membranes as well as nucleic acids. The study of the effect of *Thymus vulgaris* and thymol on the biofilm formation of *Candida albicans* and *C. tropicalis* showed a significant reduction in biofilm formation, leading to the disaggregation and deformity of *C. albicans* biofilm cells, and reduced hyphae formation in *C. tropicalis*. There was also observed synergy between *T. vulgaris*/thymol and fluconazole against both planktonic and biofilm growth of *Candida* species [80]. Qu et al. [81] found that the application of carvacrol in a dose-dependent manner across concentrations of 0, 50, 100, and 200 µg/mL causes significant inhibition of *A. flavus* spore germination, mycelial growth, AFB1 production, and ergosterol production in mycelia. The study carried out by da Silva Bomfim et al. [75] confirmed that *R. officinalis* EO, mainly consisting of 1,8-cineole (52.2%), camphre (15.2%), and alpha-pinene (12.4%), applied at 150 μg/mL, significantly reduced the mycelial growth of *Fusarium verticilioides*; at 300 μg/mL, significant morphological changes were visualized by the microscope, such as the rupture of the cell wall and the leakage of cytoplasm, thus the loss of cell components. Rosemary EO, mainly composed of 1,8-cineole, camphor, and α-pinene, strongly prevented the spore germination of *F. culmorum*, *F. oxysporum,* and *P. italicum* [17]. Our rosemary EO, mainly composed of camphre (41.2%), camphene (18.1%), and α-pinene (17.4%), has strongly affected all the *Fusarium* species by fumigation technique except the *F. culmorum*. So, the antifungal effect is dependent on both the EO composition and the technique applied. It has been mentioned that EO vapors have the ability to attack the life cycle of molds at the germination stage, as well as in the phases of hyphal growth and sporulation. The inactivation of conidia in the air by EO vapors is a key process of inhibition since conidia (airborne) are stable to heat, light, and chemical compounds and are very difficult to remove. This effect was only observed in contact with EO vapors and not in liquid form [82]. In addition to their effectiveness alone, the EO mixtures may present a higher antimicrobial effect; this may be relative, as was shown in the study by Ebani et al. [83], where EOs from *Origanum vulgare*, *Satureja montana*, and *Thymus vulgaris* exhibited notable activity against the primary bacterial species responsible for canine otitis externa. When combined in a mixture, their antimicrobial effectiveness in vitro was notably boosted, inhibiting bacterial growth at remarkably MIC levels. However, it is worth noting that the essential oil from *S. montana* alone demonstrated greater sensitivity against *Malassezia pachydermatis*, indicating a potential antagonistic interaction among the three essential oils when combined.

Although the literature cites the phenolic compounds carvacrol and thymol as being responsible for the antimicrobial activity of thyme, our thyme EO contains no trace of these compounds; however, its effectiveness was remarkable compared to that of rosemary. In addition to its high inhibitory effect on the majority of tested fungi, *T. ciliatus* EO had a fungicidal effect on six molds (*A. glaucus*, *B. cinerea*, *B. aclada*, *Cl. herbarum*, *Cl. sphaerospermum*, *M. suaveolens*, and *U. chartarum*) compared to *R. officinalis* (*A. glaucus*, *Cl. Sphaerospermum*, and *M. suaveolens*). This fungicidal effect was dose-dependent (Table 4). This shows that there is a synergistic effect of all the components of the EO on its antioxidant and antimicrobial power.

## 3. Materials and Methods

### 3.1. Plant Material and Essential Oil Extraction 

The plant material, composed of *Thymus ciliatus* (Desf.) Benth. growing wild in the mountainous region of Djbel Messaâd (Bou-saâda) (voucher specimen:TC2369QS28DM) and the spontaneous *Rosmarinus officinalis* L. of Hammam-Dalâa mountains in M’sila, Algeria (voucher specimen: RO2314QS28KA), was collected in March 2016 at the flowering stage. After being air-dried in the shade at room temperature, the aerial parts were stored in clean paper bags until use. Essential oil extraction was carried out by subjecting 100 g of each plant material to hydrodistillation for 3 h with 1000 mL of distilled water using a Clevenger-type apparatus. The extracted oils were collected and dried over anhydrous sodium sulfate, then stored in sealed glass vials at −4 °C until use.

### 3.2. The Methanol Extract Preparation

Thirty grams of the powdered plant material were subjected to Soxhlet extraction using 300 mL of methanol at 40 °C for 8 h. After filtration through Whatman paper, the methanol extract was concentrated under reduced pressure on a rotary evaporator until dryness and then weighed. The extracts were kept in the dark at 4 °C until use.

### 3.3. GC- and GC-MS Analyses and Identification of the EOs Components

Hewlett-Packard gas chromatograph mod. 5890, fitted with a flame ionization detector (FID) and linked to an electronic integrator, was used for gas chromatographic (GC) studies. The analytical parameters used for the GC-FID analyses were as follows: a ZB-5 capillary column (30 m × 0.25 mm i.d. × 0.25 μm film thickness); helium as the carrier gas; injection in split mode (1:50); and injector and detector temperatures of 250 and 280 °C, respectively. The oven’s temperature was set to rise by 2 °C per minute from 40 °C to 300 °C. Using a Hewlett-Packard mass spectrometer model 5971A with an ionization voltage of 70 eV, an electron multiplier of 1700 V, and an ion source temperature of 180 °C, gas chromatography-mass spectrometry (GC-MS) was conducted on the same gas chromatograph. Mass spectra data were obtained in the scan mode within the *m*/*z* range of 40–400. The above-mentioned gas chromatographic conditions were applied. The GC retention index (in relation to C9–C22 n-alkanes on the ZB-5 column), computer matching of spectral MS data with the Wiley 275 library [84], fragmentation pattern comparison with published literature, and, if feasible, co-injections with genuine samples were used to determine the identity of the components.

### 3.4. Determination of the Total Phenolic and Flavonoid Contents 

Polyphenolic content was determined as described in Wong et al. [85]. One hundred microliters of the extract were mixed with 2.5 mL of the Folin–Ciocalteu reagent (10× dilutions). After a 5 min reaction, 2.5 mL of Na_2_CO_3_ solution (7.5% *w*/*v*) was added and allowed to stand for 2 h. The absorbance was measured at 765 nm in a spectrophotometer. The total polyphenols were expressed as μg of GAE/mg dry extract by using an equation obtained from a standard gallic acid linear calibration curve.

Total flavonoid content was determined as in Sarikurkcu et al. [86], with some modifications. One milliliter of 2% aluminum trichloride (AlCl_3_) in methanol was mixed with the same volume of the diluted essential oil or extract solutions. Absorbance values of the samples were determined at 415 nm after a 15 min duration against a blank sample consisting of methanol (1 mL) and extract (1 mL) without AlCl_3_. Quercetin was used as a reference compound to produce the standard curve, and results were expressed as μg of QE/mg of dry mass.

### 3.5. Antioxidant Activity

#### 3.5.1. DPPH Radical Scavenging Activity Assay 

The DPPH free radical scavenging activity was performed as in Hazzit et al. [52]. From the methanolic concentration series, the essential oil or methanol extract (50 µL) was added to 2 mL of a 0.004% methanolic solution of DPPH. The absorbance was measured at 517 nm against the control (methanol without essential oil or methanol extract) after a 20 min dark incubation period at room temperature. A positive control in the form of BHT was utilized. This formula was used to determine the inhibition of the DPPH free radical:

% Inhibition = [(A_control_ − A_sample_)/A_control_] × 100

where A_control_ is the absorbance of the control sample that contained all reagents except for the tested sample and A_sample_ is the absorbance of the tested sample. Percentages of inhibition were plotted against concentrations of essential oil or methanol extract to calculate the concentration providing 50% inhibition (IC_50_).

#### 3.5.2. β-Carotene Bleaching Assay

As described by Shukla et al. [87], 0.5 mg of β-carotene in 1 mL chloroform, 25 µL of linoleic acid, and 200 mg of Tween 40 were combined to make a stock solution of β-carotene-linoleic acid. In a rotatory evaporator (40 °C), the chloroform was evaporated. After that, 100 mL of distilled water was added, and the mixture was agitated. Then, 2.5 mL aliquots of the β-carotene-linoleic acid emulsion were added to test tubes that held 350 µL of different plant ME or EO methanolic concentrations. At 470 nm, the absorbance was measured instantly. The test tubes were kept with blanks in a 50 °C hot water bath, with BHT serving as a positive control and methanol serving as a negative control instead of the extract. After incubation for 120 min, the absorbance was measured once again. The inhibition percentages were averaged after each test was run three times. The following formula was used to obtain the values for antioxidant activity (inhibition percentage, or I%):

I% = [(A_t_ − C_t_)**/**(C_0_ − C_t_)] × 100

where C_0_ is the control’s absorbance at t = 0 min, and A_t_ and C_t_ are the sample’s and control’s absorbances at 120 min, respectively. The results are displayed as IC_50_ values (µg/mL), which indicate the concentration needed to inhibit β-carotene bleaching by 50%.

#### 3.5.3. Reducing Power Assay

This test was performed as described by Esmaeili and Sonboli [88]. After mixing 0.75 mL of potassium hexacyanoferrate [K_3_Fe(CN)_6_] (*w*/*v*, 1%) and 0.75 mL of phosphate buffer (0.2M, pH 6.6) with the ME, EO, or BHT at different concentrations (in methanol), the mixture was incubated for 20 min at 50 °C in a water bath. Next, 0.75 mL of trichloroacetic acid (TCA) solution (10%) was added to end the reaction, and the mixture was centrifuged for 10 min at 3000 rpm. The supernatant (1.5 mL) was mixed with 1.5 mL of distilled water and 0.1 mL of a 0.1% *w*/*v* ferric chloride (FeCl_3_) solution for 10 min. The reducing power was determined by measuring the absorbance at 700 nm. The EC_50(RP)_ value, which represents the concentration of extract at which absorbance is 0.5, was calculated for ME, EO, and BHT.

### 3.6. Antifungal Activity

#### 3.6.1. Fungal Strains

Thirty-two mold species were used in this study; Eight species (*Aspergillus flavus*, *A. niger* ATCC16404, *A. ochraceus* ATCC28947, *A. parasiticus*, *Fusarium oxysporum*, *Penicillium citreonigrum*, *P. frequentens*, and *Ulocladium chartarum*) were obtained from Dipartimento Scienze del Farmaco e dei Prodotti per la Salute, Università di Messina, Contrada Annunziata, I-98168 Messina, Italy; 7 species (*Aspergillus flavus* NRRL3251, *A. parasiticus* CBS100926, *Fusarium culmorum*, *F. graminearum*, *F. moniliforme*, *F. oxysporum* f. sp. *lini*, and *F. proliferatum*) were obtained from Laboratoire de Biologie des Systèmes Microbiens (LBSM), Ecole Normale Supérieure de Kouba, Algérie; and 17 species were isolated from decayed fruits and vegetables and identified according to the identification technique of Pitt and Hocking [89] based on the culture of molds on Czapek Yeast Extract Agar (CYA), Malt Extract Agar (MEA), and 25% Glycerol Nitrate Agar (G25N) and keys for determination (colony diameter, color and texture, and microscopic characteristics: hyphae and conidiophore appearance, size and shape of vesicles, metulae, phialides, and conidia, etc.) described elsewhere [90,91,92,93]. All molds were cultured on PDA and then kept at 4 °C until use. 

#### 3.6.2. Antifungal Activity Assays

##### Effect of Plant Powder on the Mold Mycelial Growth

The effect of the plant powder was carried out as described by Ameziane et al. [25]. Ten grams of the plant powder was added to 100 mL of melted PDA medium at 40 °C. The resulting suspension was stirred for 10 min, autoclaved for 15 min at 121 °C, and dispensed into Petri plates 9 cm in diameter. Mold grown on PDA without plant powder was used as the control. The prepared plates were inoculated aseptically with 6-mm-diameter disks of the test fungi taken from the actively growing edge of one 7-day-old culture and incubated at 25 °C for 7 days. Radial growth was determined by measuring colony size along two perpendicular axes and the test was performed in triplicate. The antifungal effect was expressed as the percentage of mycelial growth inhibition (MGI%) calculated according to the formula:

MGI% = [(control diameter − test diameter)/control diameter] × 100(1)

##### Agar-Well Diffusion Method

The ME was screened for its antifungal activity using the well-plate diffusion method [94]. Wells (Ø 8 mm) were made at three locations per Petri plate containing 20 mL of PDA (each plate formed a triplicate test). The wells were then filled each with 20 µL of the solvent extract at a concentration of 0.1 g·mL^−1^. Control plates consisted of wells filled with the solvent. A mycelial disc (Ø 6 mm) was taken from the periphery of an actively growing agar culture (7 days old) and placed at the center of the dish containing the extract. Inoculated Petri plates were incubated at 25 °C in darkness and observations were recorded daily up to the 7th day. Mycelial growth inhibition (MGI %) was calculated by Formula (1).

##### Fumigation Bioassay

This bioassay was conducted according to Feng et al. [95]. A mycelial disc (Ø 6 mm) from the 7-day-old culture was put in the middle of a 90-mm Petri dish of PDA. Various amounts of EO (5, 10, and 15 µL) were added to a sterilized filter paper disc (Ø 9 mm), which was then placed on the cover of the dish that was maintained upside-down. Rapid parafilm sealing and 25 °C incubation were applied to the dishes. Distilled water was applied to the controls instead of the EO. The fungal development was noted every day, up until the seventh day. The percentage of radial growth inhibition in comparison to the control was used to determine growth inhibition as above (1).

##### Contact Bioassay

The experiment was conducted according to the method of Marandi et al. [96]. The PDA medium was autoclaved and cooled to approximately 45 °C. The EO was aseptically added to achieve the final different concentrations of 500, 1000, and 1500 µL/L in the molten PDA containing Tween 80 (0.5% *v*/*v*). The resulting media were immediately dispensed (15 mL) into sterilized Petri plates (Ø 9 cm) and then inoculated at the center with 6 mm plugs from the 7-day-old fungal cultures. In the control, water was used instead of the EO. Inoculated Petri plates were incubated at 25 °C in darkness, and observations were recorded daily up to the 7th day. Three replicates were used per treatment. Mycelial growth inhibition (MGI %) was calculated by Formula (1).

### 3.7. Statistical Analysis

All experiments were conducted in triplicate and data are expressed as mean ± SD. The analysis of variance (ANOVA) and Tukey’s multiple comparison were considered significant at *p* < 0.05. The statistical analysis was carried out with GraphPad Prism (version 6.05; GraphPad Software Inc., Boston, MA, USA).

## 4. Conclusions

Food safety continues to be a major concern for consumers, governments, and the food industry around the world. Although synthetic antimicrobials are approved in many countries, the recent trend has been for the use of natural preservatives, which requires the exploration of alternative sources of safe, effective, and acceptable natural preservatives. Many plant extracts have antimicrobial activity against a range of bacteria, yeasts, and fungi, but variations in the quality and quantity of their bioactive constituents are a major disadvantage to their industrial uses.

It can be concluded that the studied plants have optimal EO yields compared to those of the Mediterranean region. Their MEs are rich in polyphenols and flavonoids. *Rosmarinus officinalis* EO presents camphor, camphene, α-pinene, and 1,8-cineole as major components, while *T. ciliatus* has α-pinene, myrcene, β-caryophyllene, germacrene D, and β-pinene as the main components. The plant MEs showed high power in terms of antioxidant activity, particularly as radical scavengers. In terms of the antifungal activity of the plants studied, all the concentrations applied by the different methods reduced the growth of the tested molds. The application of methanol extract was the least effective of the applied methods. The application of the powdered plant reduced growth to acceptable levels. The EO was more effective, and that of *T. ciliatus* was the strongest, given that more than 50% of the tested fungi were sensitive to it. Remarkably, there were no residues of thymol or carvacrol in the *T. ciliatus* EO. These components are tightly linked to the *Thymus* species and are known to be responsible for their antibacterial activity. Therefore, our thyme presented an antifungal characteristic independent of these two compounds known in the other species. Additionally, the EOs given in varying amounts were fungicidal on six fungi: *U. chartarum*, *A. glaucus*, *B. cinerea*, *B. aclada*, *Cl. herbarum*, *C. sphaerospermum*, and *M. suaveolens*. Future research should focus on the effectiveness of different EOs in various food matrices. The synergy between different EOs and other compounds, as well as the application of other processing techniques, should be studied before being applied in the commercial field.

## Figures and Tables

**Table 1:** Chemical composition of *R. officinalis* and *T. ciliatus* essential oils.

			Class/Compound	*R. officinalis*	*T. ciliatus*
N° ^a^	RI Exp, ^b^	RI Lit, ^c^	Monoterpene Hydrocarbons	43.97	53.11
1	922	926	tricyclene	0.11	0.05
2	927	930	α-thujene		2.01
3	936	939	α-pinene	17.49	22.18
4	951	954	camphene	18.14	0.60
5	955	952	fenchene		0.08
6	972	975	sabinene		0.40
7	976	979	β-pinene	0.51	7.73
9	987	990	myrcene	0.42	13.13
10	1001	1002	α-phellandrene	0.11	0.13
11	1015	1017	α-terpinene	0.31	0.28
12	1023	1024	*p*-cymene	2.45	2.11
13	1029	1029	limonene	3.98	3.32
14	1036	1037	*cis*-ocimene		t
15	1046	1050	*trans*-ocimene		0.35
16	1059	1060	γ-terpinene	0.22	0.57
17	1088	1089	terpinolene	0.23	0.17
			**Oxygenated Monoterpenes**	**50.09**	**7.16**
18	1032	1031	1,8-cineole	4.90	2.58
19	1081	1086	*trans*-linalool oxide		0.21
20	1099	1096	linalool		2.29
21	1122	1121	exo-Fenchol	0.12	
22	1152	1146	camphor	41.22	0.76
23	1166	1164	pinocarvone		t
24	1169	1169	borneol	2.55	0.12
25	1179	1177	terpinen-4-ol		0.41
26	1192	1188	α-terpineol	1.18	0.71
27	1197	1195	myrtenol		0.08
28	1290	1289	bornyl acetate	t	
29	1298	1290	thymol	t	
30	1304	1299	carvacrol	0.12	
			**Sesquiterpenes**	**2.97**	**39.33**
31	1375	1375	ylangene		0.18
32	1379	1376	α-copaene	0.21	0.88
33	1388	1388	β-bourbonene		1.15
34	1388	1393	β-cubebene		0.61
35	1424	1419	β-caryophyllene	0.63	10.21
36	1433	1434	α-*trans*-Bergamotene		
37	1434	1432	β-copaene		0.82
38	1440	1441	aromadendrene		0.09
39	1445	1450	*cis*-muurola-3,5-diene		t
40	1451	1453	*trans*-muurola-3,5-diene		0.26
41	1455	1454	α-humulene		0.91
42	1458	1456	β-*trans*-farnesene	t	0.51
43	1482	1479	γ-muurolene		1.14
44	1485	1484	α-amorphene	0.32	
45	1486	1484	germacrene D	0.12	9.90
46	1486	1486	β-selinene		0.14
47	1490	1490	*trans*-muurola-4(14),5-diene		0.52
48	1495	1495	bicyclogermacrene		0.42
49	1495	1495	γ-Amorphene	0.11	
50	1498	1496	valencene	t	
51	1500	1500	α-muurolene		0.27
52	1505	1505	β-bisabolene	0.10	0.18
53	1512	1513	γ-cadinene	0.11	
54	1513	1512	δ-amorphene		1.51
55	1520	1523	δ-cadinene	0.32	2.16
56	1535	1534	*trans*-cadina-1(2),4-diene		0.05
57	1538	1538	α-cadinene	t	0.06
58	1548	1545	α-calacorene	t	t
59	1560	1549	elemol		1.52
60	1588	1578	spathulenol		t
61	1597	1583	caryophyllene oxide		0.70
62	1627	1631	eremoligenol		0.21
63	1649	1632	γ-eudesmol		0.51
64	1654	1654	α-cadinol		2.1
65	1661	1656	α-muurolol		0.04
66	1664	1660	β-eudesmol		1.11
67	1676	1663	α-eudesmol		1.17
68	1691	1685	α-bisabolol	1.15	
			**Others**		**0.05**
8	985	984	3-octanone		0.05
			**Monoterpene hydrocarbons**	**43.97**	**53.11**
			**Oxygenated monoterpenes**	**50.09**	**7.16**
			**Sesquiterpenes**	**2.97**	**39.33**
			**Others**		**0.05**
			**Total**	**97.03**	**99.65**

^a^ The numbering refers to elution order, t = trace, <0.05%. ^b^ Retention index (RI) relative to standard mixture of n-alkanes on SPB-5 column; ^c^ Literature Retention Index (RI).

**Table 2:** Total polyphenol and flavonoid contents of MEs and EOs of *R. officinalis* and *T. ciliatus*. The results are expressed as means (*n* = 3) ± SD.

Extract/Standard	Polyphenols (µg GAE/mg)	Flavonoids (µg QE/mg)
*T. ciliatus* ME (MET)	81.97 ± 1.19 ^a^ *	48.01 ± 0.99 ^a^ *
*R. officinalis* ME (MER)	127.1 ± 2.40 ^b^	38.61 ± 0.75 ^a^
*T. ciliatus* EO (EOT)	13.24 ± 0.09 ^c^	0.02 ± 0.01 ^b^
*R. officinalis* EO (EOR)	7.81 ± 0.41 ^d^	0.01 ± 0.00 ^b^

* The means followed by the same superscript letter in the same column are not significantly different (*p* < 0.05) according to Tukey’s multiple comparisons test.

**Table 3:** Antioxidant activity of the tested plant extracts and standard antioxidants is expressed in IC_50_/EC_50_ values (μg/mL) based on the DPPH, β-carotene, and reducing power (RP) tests. The results are expressed as means (*n* = 3) ± SD.

Extract/Standard	DPPH IC_50_ (µg/mL)	β-Carotene/Linoleic Acid IC_50_ (µg/mL)	RP EC_50_ (µg/mL)
MET	17.03 ± 0.16 ^a^ *	165.70 ± 3.82 ^a^ *	53.86 ± 1.68 ^a^ *
MER	13.43 ± 0.14 ^b^	39.01 ± 2.16 ^b^	15.03 ± 1.43 ^b^
EOT ^#^	3.82 ± 0.10 ^c^	0.96 ± 0.14 ^c^	0.37 ± 0.01 ^c^
EOR ^#^	3.37 ± 0.05 ^c^	0.78 ± 0.01 ^c^	0.69 ± 0.01 ^d^
BHT	21.91 ± 0.21 ^e^	0.58 ± 0.03 ^d^	5.37 ± 0.25 ^e^

^#^ The values are represented by (μL/mL). * The means followed by the same superscript letter in the same column are not significantly different (*p* < 0.05) according to Tukey’s multiple comparisons test.

**Table 4:** Fungicidal effect of *R. officinalis* and *T. ciliatus*: fully killed molds, according to the technique and concentration applied.

	Contact Bioassay (Incorporation) (µL/L)						Fumigation Bioassay (µL)					
Fungal Isolates	*R. officinalis*			*T. ciliatus*			*R. officinalis*			*T. ciliatus*		
	500	1000	1500	500	1000	1500	5	10	15	5	10	15
*A. glaucus*				F	F	F		F	F	F	F	F
*B. cinerea*					F	F					F	F
*B. aclada*												F
*Cl. herbarum*						F						
*Cl. sphaerospermum*									F			
*M. suaveolens*			F			F						
*U. chartarum*												F

F: Fungicidal effect.

## Data Availability

Data are contained within the article.
